# Alcohol drinking as a mediator of the influence of smoking prevalence on second-hand smoke exposure in workplaces: a mediation analysis

**DOI:** 10.1186/s13011-020-00307-0

**Published:** 2020-09-03

**Authors:** Haoxiang Lin, Chun Chang, Zhao Liu

**Affiliations:** 1 Department of Social Medicine and Health Education, School of Public Health, Peking University Health Science Center, Postal address: 38. Xueyuan Rd, Haidian District, Beijing, China; 2 Tobacco Medicine and Tobacco Cessation Center, China-Japan Friendship Hospital, Postal address: No.2 Yinghuadongjie, Chaoyang District, Beijing, China

**Keywords:** Alcohol, Smoking, Second-hand smoke

## Abstract

**Objective:**

Both alcohol drinking and second-hand smoke (SHS) exposure have shown a strong association with smoking behaviour. This study uses mediation analysis to clarify the process underlying the relationship between smoking prevalence and SHS exposure and the extent to which this relationship is mediated by alcohol use in the workplace.

**Method:**

A total of 14,195 employees from 81 companies in China participated in this survey. Mediation analysis was used to establish the mediation effect of alcohol drinking. Structural equation modelling (SEM) was used to evaluate the role of alcohol drinking when a smoke-free (SF) workplace policy was imposed.

**Results:**

For males, approximately 6.3% of the effects on SHS exposure were mediated through the channel of alcohol use. For females, this channel mediated 14.1% of the total effects. SF policy was negatively associated with smoking prevalence and SHS exposure. The indirect effect of the SF policy on reducing SHS exposure by controlling smoking behaviour was significant. For smokers, worksite smoking bans were negatively associated with the smoking amount and drinking behaviour, and the indirect effect of this policy on promoting smokers’ intention to quit by reducing the smoking amount and controlling drinking behaviour was significant.

**Conclusion:**

This study confirmed that alcohol drinking is among the channels that mediate the association between smoking prevalence and SHS exposure in workplaces. Our results also find a spillover effect of SF workplace policy and call for effective interventions for alcohol use, which may influence the outcomes of SF policy implementation.

## What this paper adds

Using the ABWMC data, this study finds that alcohol drinking is among the channels that mediate the association between smoking prevalence and SHS exposure in workplaces. The SF workplace policy not only can reduce SHS exposure but also has an indirect effect on promoting quitting intention by controlling drinking behaviour and reducing the smoking amount.

## Introduction

Both alcohol drinking and second-hand smoke (SHS) exposure have shown a strong association with smoking behaviour. Smoking and alcohol drinking have a positive correlation, and a higher level of smoking prevalence in an area is related to a higher level of SHS exposure than that reported by non-smokers [1–3]. However, the extent to which adjustment for alcohol use modifies the association between smoking and SHS exposure remains uncertain. Most studies that have investigated the effects of alcohol drinking on smoking-related behaviours have relied on a general view, and few have assessed the effects in particular settings, such as workplaces [4, 5]. The workplace is among the most common settings where individuals report suffering from SHS exposure, which may be particularly deleterious to health [6].

Because of the sustained increase in working hours in the past half century and the high prevalence of smoking and alcohol use in China, the link between smoking and drinking behaviour and workplace SHS exposure has become increasingly important for public health and policy makers. For example, over the past three decades, there has been a > 4-fold increase in per capita alcohol consumption following the rapid economic development that entailed cultural and behavioural changes [7]. A national representative study showed that 34.3% of participants in China described themselves as regular drinkers (54.6% for males and 13.3% for females) [8], and those who drank more also smoked more cigarettes [9]. In addition, a very recent study found that the prevalence of alcohol consumption was 45.84% for young Chinese people (drinkers’ referred to those who have either drunk more than half a cup of alcohol at least once in the past 12 months or those who have drunk any quantity of alcohol at least twice in the past 12 months), suggesting an increase in alcohol use among young people [10].

Another problem is drinking at work. Alcohol can reduce cognitive and behavioural performance. Nevertheless, a large number of employees report consuming alcohol before work or during work, working under the influence of alcohol, or working with a hangover [11]. A large number of studies have investigated the huge cost of this behaviour on employees and society. However, most of these studies focused on medical problems and performance, and none of them described the association between workplace smoking-related behaviours and policy [12, 13].

Confounding or mediator variables in smoking-related studies are usually controlled by multivariate linear regression or logistic regression depending on the objective of the study [14, 15]. Mediation analysis is a statistical procedure to examine any possible mediating variables. A mediation effect occurs when a third variable carries the influence of a given independent variable on a given dependent variable. We hypothesized that alcohol drinking acts as a mediator in the relationship between workplace smoking prevalence and SHS exposure. Therefore, we used mediation analysis to clarify the process underlying the relationship between smoking and SHS exposure and the extent to which this relationship is mediated by alcohol use.

This study further explores the role of alcohol consumption when a smoke-free (SF) workplace policy is imposed and the mechanisms that promote smokers’ intention to quit. To our knowledge, our study is the first to investigate the mediating role of alcohol use on the relationship between smoking and SHS exposure in the workplace.

## Method

### Data

The data were derived from the 2018 Asia Best Workplace Mainland China (ABWMC) programme, which aims to support companies in building a healthy workplace. The ABWMC programme was designed by Peking University and organized by the American International Assurance Co. All companies may voluntarily join the programme and are free to withdraw. The inclusion criteria were as follows: (1) registered legal companies in China; (2) agreement to participate in the programme; and (3) at least 100 workers who are full-time employees.

Data were collected by employee questionnaires. The questionnaires were designed by Peking University and accessed via an online link through a survey company, Ipsos Inc. The questionnaire consisted of 50 items, including demographic and sociological information, payment and welfare information, individual health literacy and lifestyle information, smoking-related behaviour and quitting intentions, and disease and sick leave information.

The human resource departments of each company delivered the questionnaires to all employees. When employees first opened the link, content related to informed consent was shown, and the employees were able to choose whether to complete the questionnaire or quit. We considered them to have agreed to participate if they submitted the questionnaire through the link. The self-check function of the online survey system automatically identified missing data, logical errors and illegal characters. All participants were informed that the research team would analyse the data anonymously.

Our analyses used all participants for whom the variables of interest were available, with no imputation for missing data.

### The measurement of alcohol use and smoking and quitting intention

Alcohol use was identified by a question that asked respondents, ‘How often do you drink alcohol?’ The response options were A: everyday, B: always C: sometimes, D: I never drink alcohol. These four groups were collapsed into two groups of non-drinkers (D) and drinkers (A or B or C). In this study, we also measure drinking by drinking prevalence. This referred to point prevalence when we conducted the survey.

Smoking was measured by the question, ‘Do you smoke now? A: yes, every day, B: yes, only sometimes, C: I have quit smoking, D: never.’ Participants who chose A or B were classified as smokers. In this study, we also measure smoking by smoking prevalence. This referred to point prevalence when we conducted the survey.

In the survey, participants were asked, ‘Are you going to quit smoking? A: yes, within a month, B: yes, within 6 months, C: yes, but not within 6 months, D: no plan for quitting’. Participants who chose A or B were classified as having an intention to quit.

### Measurement of SHS exposure and SF workplace policy

In the survey, participants were asked, ‘How many days do you usually suffer from SHS exposure more than 15 minutes a week in the workplace? A: almost every day, B: 4-6 days, C: 1-3 days, D: never.’ Only participants who chose D were classified as having no SHS exposure.

Although there are legal ‘recommendations’ regarding SF workplaces, mainland China does not have national legislation for either comprehensive SF public places or SF workplaces. Some companies have voluntarily banned smoking due to safety requirements and health concerns. Therefore, in this study, we used company-level SF workplace bans as a measurement of indoor smoking policies. We measured worksite SF policy by asking about smoking rules in the workplace. The response options were as follows: A: no smoking ban, B: only ban smoking in parts of indoor area, C: complete smoking ban inside building, D: I have no idea. Only the participants who chose C were classified as working in a company with a SF policy.

We controlled for several variables of individual characteristics, such as gender, age, body mass index (BMI), marital status, ethnicity, education, yearly income, chronic disease and job position.

### Data analysis

#### Mediation analysis to establish the mediating effect of alcohol drinking

To examine whether the association between smoking prevalence and SHS exposure was mediated by alcohol use, linear regression models were fitted based on the procedures outlined by Baron and Kenny [16]. The first equation regressed the mediator on the independent variable. The second equation regressed the dependent variable on the independent variable. The third equation regressed the dependent variable on both the independent variable and mediator.

The present study utilized the following criteria to establish mediation [17]:

1. The independent variable (smoking) should be significantly related to the mediator (alcohol drinking) and the dependent variable (SHS exposure).
2. The mediator (alcohol drinking) must be significantly related to the dependent variable (SHS exposure).
3. The association between the independent variable (smoking) and the dependent variable (SHS exposure) must be attenuated when the mediator (alcohol use) is included in the regression model.

We then performed Sobel tests to estimate how much of the effect was mediated through the channel of alcohol use. The Sobel test is widely used to investigate the size and significance of indirect relationships. It is basically a specialized t-test used for examining whether the effect of the independent variable has a statistically significant reduction after the mediator variables are included [18]. In addition, as a supplemental method, we tested the mediation effects using a bootstrap test. As the result was almost the same, we report only the Sobel test results. We conduct the mediation analysis for male and female separately, because of significant differences in cigarette smoking by gender among adults in China (The 2018 China Adult Tobacco Survey shows 50.5% of males and 2.1% of females are smokers) [19].

#### Structural equation modelling (SEM) to evaluate the role of alcohol drinking when SF workplace policies are imposed

We applied a structural equation modelling (SEM) approach to test two hypothesized models. For the first model, we used a full sample to test the role of alcohol consumption in the pathways between SF workplace policy and SHS exposure. For the second model, we added smoking amount and quitting intention to the model and tested the role of regular alcohol drinking in the pathways between workplace SF policy and quitting intention among smokers. The SEM approach can be used to test overall models rather than individual coefficients, incorporating multiple dependents as well as mediating variables [20, 21].

We used the following model fit statistics that have proven to be meaningful in SEM [20, 21]:

Bentler’s comparative fit index (CFI): recommended> 0.95;

Tucker-Lewis index (TLI): recommended> 0.95;

Root mean square error of approximation (RMSEA): < 0.06.

We used AMOS 24.0 for SEM and STATA 14.0 for mediation analysis.

## Results

The total number of participants was 14,195 employees from 81 companies in mainland China. Regarding the companies, 51.9% were private companies, 32.9% were foreign companies, 7.6% were state-owned companies, 6.3% were joint ventures, and 1.3% were other companies.

Table 1 presents descriptive statistics for the targets of the analysis. The mean age was 31.6 years (SD = 7.27). Most respondents achieved college graduation. The point smoking prevalence was much higher among males (40.5%) than among females (4.6%). Ten thousand one hundred seven eight participants (71.7%) reported working under SF policies. Overall, 51.7% of the respondents reported any alcohol use (point prevalence), and 67.1% of smokers reported that they had an intention to quit.

**Table 1:** Demographics and key variables in study population

Demographics	Male (n,%)	Female (n,%)	Overall (n, %)
**Age**			
16–29	2641 (41.2)	3587 (46.1)	6228 (43.9)
30–39	2704 (42.2)	3228 (41.5)	5932 (41.8)
40–49	876 (13.7)	918 (11.8)	1794 (12.6)
50 and above	187 (2.9)	54 (0.7)	241 (1.7)
Mean age (SD)	31.72 ± 7.32	31.49 ± 7.23	31.60 ± 7.27
**Marriage**			
Single	2409 (37.6)	3125 (40.1)	5534 (39.0)
Married	3939 (61.5)	4552 (58.5)	8491 (59.8)
Divorced or widowed	60 (0.9)	110 (1.4)	170 (1.2)
**Ethnicity**			
Han	6157 (96.1)	7437 (95.5)	13,594 (95.8)
Others	251 (3.9)	350 (4.5)	601 (4.2)
**Education attainment**			
Middle school or lower	233 (3.6)	243 (3.1)	476 (3.4)
High school	1005 (15.7)	1075 (13.8)	2080 (14.7)
College	4572 (71.3)	5802 (74.5)	10,374 (73.1)
Master and above	598 (9.3)	667 (8.6)	1265 (8.9)
**2017-year income**			
100 K or below	3434 (53.6)	5087 (65.3)	8521 (60.0)
100 K–150 K	1237 (19.3)	1383 (17.8)	2620 (18.5)
150 K–200 K	632 (9.9)	553 (7.1)	1185 (8.3)
200 K–300 K	592 (9.2)	421 (5.4)	1013 (7.1)
300 K or above	513 (8.0)	343 (4.4)	856 (6.0)
**Secondhand smoke exposure**			
Never	2362 (36.9)	4067 (52.2)	6429 (45.3)
1 to 3 days per week	2059 (32.1)	2364 (30.4)	4423 (31.2)
4 to 6 days per week	539 (8.4)	442 (5.7)	981 (6.9)
Every day	1448 (22.6)	914 (11.7)	2362 (16.6)
**BMI**			
< 18.5 (underweight)	336 (5.2)	1284 (16.5)	1620 (11.4)
18.5–24.9 (normal weight)	4138 (64.6)	5084 (65.3)	9222 (65.0)
≥ 25.0 (overweight and obesity)	1934 (30.2)	1419 (18.2)	3353 (23.6)
**Workplace SF policy**			
Yes	4514 (70.4)	5664 (72.7)	10,178 (71.7)
No	1894 (29.6)	2123 (27.3)	4017 (28.3)
**Smoking**			
Yes	2593 (40.5)	362 (4.6)	2955 (20.8)
No	3815 (59.5)	7425 (95.4)	11,240 (79.2)
**No. of cigarettes smoked per day**			
1–9	467 (28.1)	82 (63.1)	549 (30.7)
10–19	799 (48.1)	39 (30.0)	838 (46.8)
20 and above	395 (23.8)	9 (6.9)	404 (22.6)
Mean age (SD)	12.66 ± 6.81	8.25 ± 6.58	12.34 ± 6.88
**Smoking quitting intention**			
Yes	1114 (67.1)	87 (66.9)	1201 (67.1)
No	547 (32.9)	43 (33.1)	590 (32.9)
**Alcohol drinking**			
Yes	4499 (70.2)	2846 (36.5)	7345 (51.7)
No	1909 (29.8)	4941 (63.5)	6850 (48.3)
**Job position**			
Not administrative	3906 (61.0)	5552 (71.3)	9458 (66.6)
Administrative	2502 (39.0)	2235 (28.7)	4737 (33.4)
**Night-shift duty**			
Yes	1628 (25.4)	1323 (17.0)	2951 (20.8)
No	4780 (74.6)	6464 (83.0)	11,244 (79.2)
**Chronic disease**			
Yes	1924 (30.0)	1965 (25.2)	3889 (27.4)
No	4484 (70.0)	5822 (74.8)	10,306 (72.6)
**Total**	6408 (45.1)	7787 (54.9)	14,195 (100)

Note: **p < 0.01

Model controlled for age, ethnic, BMI, marriage, education attainment, yearly income, chronic disease, sleep duration, position and night-shift duty

### Mediation analysis

**Fig. 1:**
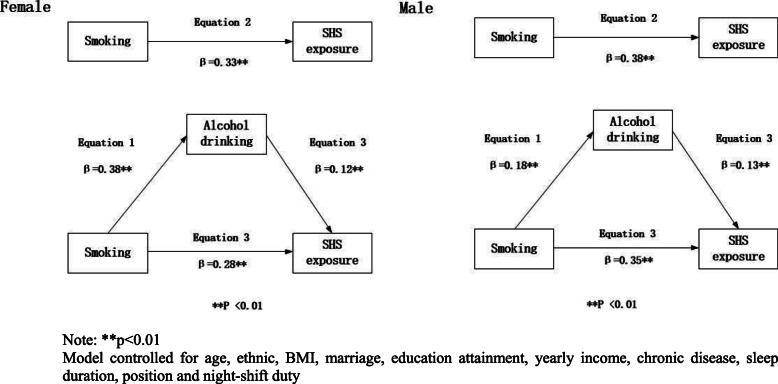
Alcohol drinking mediation models of the relationship between smoking and SHS exposure

We tested the mediating role of alcohol consumption in the relationship between smoking and SHS exposure (Fig. 1). In the first regression equation, smoking was positively associated with alcohol consumption (*P* < 0.01). In the second equation, smoking was also positively associated with SHS exposure (*P* < 0.01). Finally, in the third equation, when smoking and alcohol consumption were simultaneously included in the model, alcohol consumption and smoking were positively associated with SHS exposure (P < 0.01). The association between smoking and SHS exposure was attenuated in both females and males. These results suggest that the effect of smoking on SHS exposure was partially mediated by alcohol use.

We conducted the Sobel test for both males and females and report the results in Table 2. Alcohol use showed a confirmed role of mediation. For males, approximately 6.3% of the effects on SHS exposure were mediated through the channel of alcohol use. For females, this channel mediated 14.1% of the total effects.

**Table 2:** Sobel test of mediation for Alcohol drinking from smoking to SHS exposure

Sobel test of mediation for drinking	Total effect	Direct effect	Indirect effect	Proportion of total effect that is mediated
**Model 1**				
Male: Smoking→SHS exposure	0.408**	0.382**	0.026**	**6.4%**
Female: Smoking→SHS exposure	0.359**	0.310**	0.049**	**13.6%**
Overall: Smoking→SHS exposure	0.405**	0.360**	0.045**	**11.1%**
**Model 2**				
Male: Smoking →SHS exposure	0.386**	0.362**	0.024**	**6.3%**
Female: Smoking→SHS exposure	0.340**	0.293**	0.048**	**14.1%**
Overall: Smoking→SHS exposure	0.376**	0.334**	0.04**	**10.6%**

Note: **p < 0.01

Model 1-we only included independent variable (Smoking), dependent variable (SHS exposure) and mediate variable (Alcohol drinking) in the model

Model 2-Except independent, dependent and mediate variables, we also included control variable (age, ethnic, BMI, marriage, education attainment, yearly income, chronic disease, sleep duration, position and night-shift duty) in the model

### The role of alcohol drinking when SF workplace policies are imposed

**Fig. 2:**
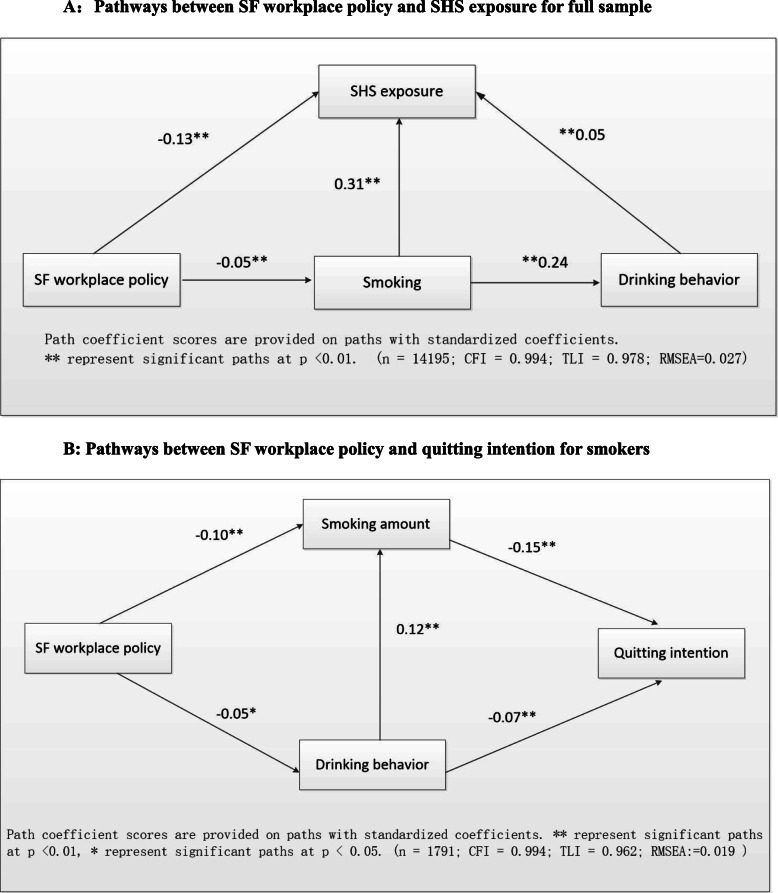
Model of pathways between SF policy to SHS exposure and quitting intention. A:Pathways between SF workplace policy and SHS exposure for full sample. B: Pathways between SF workplace policy and quitting intention for smokers

Figure 2a shows the standardized coefficients with the full sample model when the SF workplace policy was imposed. A SF policy was negatively associated with smoking (Coef. = − 0.05, *p* < .01) and SHS exposure (Coef. = − 0.13, p < .01). The indirect effect of the SF policy on reducing SHS exposure by controlling smoking behaviour was significant. However, we did not identify a direct effect of the SF workplace policy on reducing drinking behaviour in the full sample results.

For smokers (Fig. 2b), a worksite smoking ban was negatively associated with the smoking amount (Coef. = − 0.10, *p* < .01) and drinking behaviour (Coef. = − 0.05, *p* < .05), and the indirect effect of this policy on promoting smokers’ intention to quit by reducing the smoking amount and controlling drinking behaviour was significant.

## Discussion

To the best of our knowledge, this is the first study to test the mediating role of alcohol drinking in the association between smoking and SHS exposure. This study was conducted with a large population-based sample. The results support our hypotheses.

There is consistent evidence regarding the correlation between smoking and alcohol drinking or SHS exposure. However, although smoking prevalence has traditionally been considered a predictor of SHS exposure, it has not been fully clarified whether alcohol use acts as a mediator [14, 15, 22]. Our study confirms the proven relationships between smoking and SHS exposure and clarifies the mediating role of alcohol drinking in the relationship between smoking and SHS exposure.

In addition, most prior research examining the relationship between smoking and alcohol use behaviours has used general information. These studies consistently found a positive correlation [1, 23]. However, determining whether these same associations exist for specific domains, such as the workplace, and whether associations differ based on demographic variables could provide more relevant and potentially actionable information for policy makers.

In our study, we found a lower point smoking prevalence for males (40.5%) compared to the male smoking prevalence shown in the 2018 Chinese Adult Tobacco Survey (50.5%) [19]. A possible explanation is that our participants were all from different companies. Their educational attainment was higher than that of the general population; for example, 71.3% of respondents had achieved college graduation. A large number of studies have shown an inverse association between smoking and educational attainment [20, 24, 25].

The full sample SEM model showed that SF workplace policy was associated with lower SHS exposure; however, this policy had only a limited impact on drinking behaviour. However, subgroup analysis showed different results. A SF policy can have a direct influence on smokers’ drinking behaviour, which in turn works in conjunction with the impact on the smoking amount to contribute to a positive quitting intention. Therefore, it is suggested that the objective of reducing SHS exposure can be achieved through SF policies. However, in terms of triggering stronger quitting intentions or maximizing the impact of SHS exposure reduction, companies should integrate multilevel health promotion, such as alcohol drinking intervention programmes, into their employee care system.

The model of this study has some potential public health implications as it demonstrated the potential impact of alcohol use on health behaviour and the possible relationship for tobacco control policy. On the one hand, our results indicate a spillover effect of SF workplace policy, showing that SF workplace policies are associated not only with lower SHS exposure but also with reduced drinking behaviour. Moreover, our study calls for effective interventions for alcohol use, which may influence other health policy implementation outcomes. This evidence can be used to lobby policymakers to implement an effective integrated health intervention approach.

However, this is a relatively new research topic, and we acknowledge that our findings are limited by the cross-sectional design. For example, although we have proven the existence of special relationships between smoking and SHS exposure and alcohol drinking, but the coefficients appear small to the point of being tiny (from 0.05 to 0.13), we believe our results have potential theoretical interest. Therefore, future research would benefit from using longitudinal data to determine whether there are any large and meaningful effect sizes. Future studies also need to explore other potential mediators between smoking and SHS exposure and other possible factors in the pathway between SF workplace policy and SHS exposure and quitting intention. Such as, the channel of alcohol use only mediated a maximum of 10.6% of the total effects. What are the other parts of the mechanism? Does a stricter smoking ban policy have a more significant effect on controlling drinking behaviour? These issues have strong policy implications.

This study has several limitations. First, as mentioned, since we employed a cross-sectional design, we cannot infer causality. Second, selection bias might have existed due to the self-selection of participants in the ABWMC programme by company-level decision. However, because the participants were recruited from different parts of China and belonged to different types of companies, it is believed that the overall findings are meaningful. Third, other critical and omitted variables might exist that could affect the role of alcohol consumption in the model, such as smoking harm awareness and SHS harm awareness.

## Conclusions

Using the ABWMC data, this study finds that alcohol drinking is among the channels that mediate the association between smoking prevalence and SHS exposure in workplaces. Our results also identify a spillover effect of SF workplace policy and call for effective interventions for alcohol use, which may influence SF policy implementation outcomes. Taken together, the results of this empirical analysis not only contribute to identifying the determinants of SHS exposure in workplaces but also provide further evidence regarding smoking and drinking behaviour in a developing country to add to earlier research on this topic.

## Data Availability

The data of the studies is accessible via Peking University, School of Public Health.

## Abbreviation

SHS: Second-hand smoke
SF: Smoke-free
ABWMC: Asia Best Workplace Mainland China
SEM: Structural equation modelling
BMI: Body mass index
CFI: Bentler’s comparative fit index
TLI: Tucker-Lewis index
RMSEA: Root mean square error of approximation
SD: Standard deviation
